# Extensive renal sinus lipomatosis in xanthogranulomatous pyelonephritis simulating liposarcoma

**DOI:** 10.1590/S1677-5538.IBJU.2017.0509

**Published:** 2018

**Authors:** Sabrina de Mello Ando, Raquel Andrade Moreno, Públio Cesar Cavalcante Viana, Fernando Ide Yamauchi

**Affiliations:** 1 Departamento de Radiologia, Hospital das Clínicas da Universidade de São Paulo HC-FMUSP, São Paulo, Brasil

## Abstract

Renal replacement lipomatosis is a condition characterized by varying degrees of renal parenchymal atrophy and perirenal fibrofatty proliferation secondary to chronic inflammation such as xanthogranulomatous pyelonephritis. In severe cases, imaging findings can be misdiagnosed as retroperitoneal liposarcoma.

## CASE PRESENTATION

A 63-year-old man was admitted to the hospital with generalized weakness, fever and weight loss for 6 months. Blood tests showed a creatinine level of 5.29mg/dl, an urea of 169mg/dl, a C-reactive protein of 297.7mg/L and urinalysis with leukocyturia.

**Figure 1:**
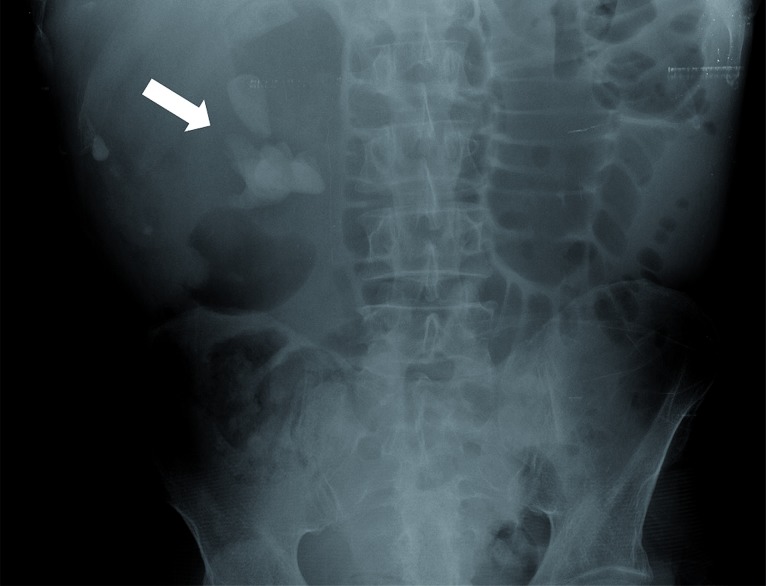
Abdominal radiography demonstrates staghorn calculus in right kidney.

**Figure 2:**
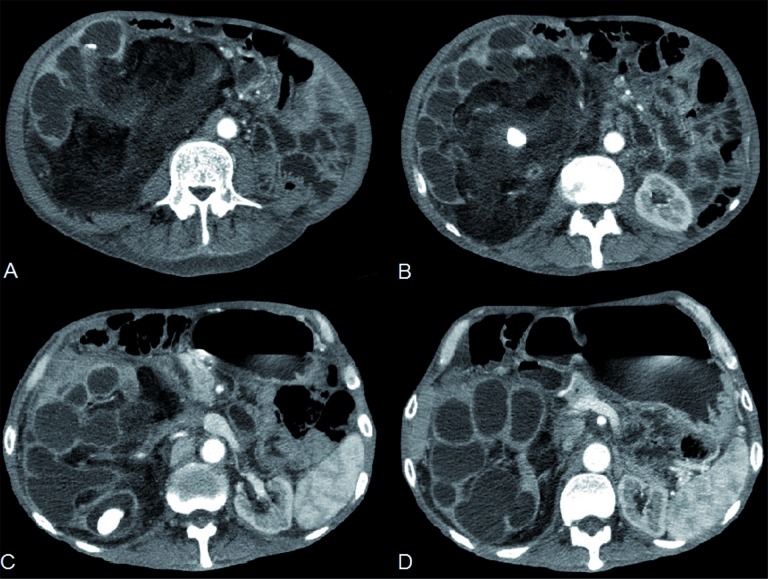
Axial post-contrast CT images show obstructive stones, dilated calyces and renal parenchymal atrophy on the right kidney. Exuberant fibrofatty proliferation in renal sinus, indicating renal replacement lipomatosis (A, B and C). calyceal dilatation with a multiloculated aspect similar to the toe pads of a bear's paw in XGP (D).

**Figure 3:**
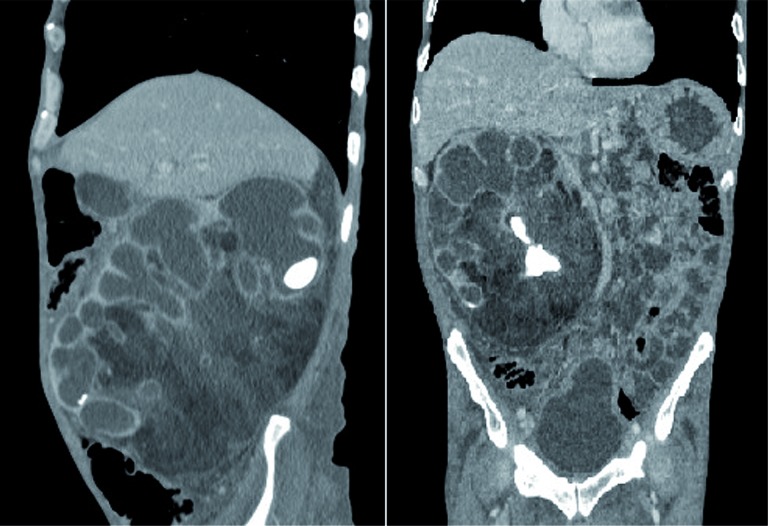
Sagittal and coronal CT images show calculi, dilated calyces and renal parenchymal atrophy and exuberant fibrofatty proliferation.

Plain abdominal radiograph demonstrated right renal staghorn calculi (Figure-1). Computed tomography (CT) images showed obstructive stone, dilated calyces and renal parenchymal atrophy with exuberant fibrofatty proliferation (Figures 2 and 3). Final diagnosis was xanthogranulomatous pyelonephritis with extensive lipomatosis.

## DISCUSSION

Renal chronic inflammation from several etiologies may induce renal parenchymal atrophy and proliferation of inflammatory and fatty cells. In severe cases of fatty proliferation and renal atrophy, the term renal replacement lipomatosis (RRL) can be used (1–4).

Xanthogranulomatous pyelonephritis (XGP) is a form of chronic inflammation, characterized by an obstructive staghorn calculous, hydronephrosis and renal atrophy (1–4). On pathology, there is destruction of renal parenchyma and replacement by lipid-laden macrophages (xanthoma cells) associated to other inflammatory cells, including plasma cells, leukocytes, and histiocytes (1–4).

Typical symptoms are nonspecific, such as flank pain, fever, fatigue, weight loss and dysuria. A palpable flank mass may be detected on physical examination. Leukocytosis and anemia are common laboratory findings and urine culture may identify Escherichia coli, Proteus mirabilis, Staphylococcus aureus, Klebsiella or Pseudomonas (4–6).

Plain radiography may demonstrate a large staghorn calculus, renal contour enlargement and, in advanced disease, obscuration of ipsilateral psoas margin (1, 5). Ultrasound usually depicts renal enlargement with dilated calyces and parenchymal destruction, renal stone and staghorn calculus (1, 4). In RRL, lipomatous tissue from renal sinus appear as an hyperechoic mass, indistinguishable from a primary retroperitoneal mesenchymal tumor (5).

Despite the findings on plain radiography and ultrasound, CT remains the best imaging modality to evaluate these conditions, not only for diagnosis but also to evaluate extension and surgical planning. An obstructive pelvic stone and calyceal dilatation with a multiloculated aspect can be observed, similar to the toe pads of a bear's paw in XGP. This appearance reflects an atrophic renal parenchyma replaced by enlarged calyces with thick content (2, 4, 6–8).

In the RRL, besides the findings of XGP, there is extensive fatty tissue within the renal sinus, hilum and perinephric space (2, 5). Those characteristics are shared with retroperitoneal liposarcoma, a rare tumor that arises from the re-troperitoneum that may occur in this region, and may impose diagnostic dilemmas (9). Since in RRL and XGP there is minimal or absent renal function on the affected kidney, nephrectomy is usually the treatment of choice (10).
